# Excessive Promoters as Silencers of Genes Horizontally Acquired by *Escherichia coli*

**DOI:** 10.3389/fmolb.2020.00028

**Published:** 2020-02-26

**Authors:** Aleksandr Bykov, Olga Glazunova, Olga Alikina, Natalia Sukharicheva, Irina Masulis, Konstantin Shavkunov, Olga Ozoline

**Affiliations:** ^1^Laboratory of Functional Genomics and Cellular Stress, Institute of Cell Biophysics of the Russian Academy of Sciences, Pushchino Scientific Center for Biological Research, Moscow, Russia; ^2^Research Institute of Molecular Pathology, Vienna Biocenter Campus, Vienna, Austria

**Keywords:** horizontal gene transfer, gene silencing, promoter islands, transcription, H-NS, error-prone mutagenesis, *gfp* reporter assay

## Abstract

Horizontally acquired genes are usually transcriptionally inactive, although most of them are associated with genomic loci enriched with promoter-like sequences forming “promoter islands.” We hypothesized that lateral DNA transfer induces local mutagenesis, accumulating AT base pairs and creating promoter-like sequences, whose occupancy with RNA polymerase and a specific silencer H-NS suppresses the transcription of foreign genes. Error-prone mutagenesis was implemented for the “promoter island” of a foreign gene *appY* and the promoter region of an inherent gene *dps*. Derivatives with changed transcriptional activity were selected using a reporter plasmid pET28_eGFP. Only one cycle of mutagenesis with negative selection suppressed the activity of the main *dps* promoter to the background level due to a single substitution in its -10 element, while positive selection gave a sequence with improved -35 element, thus testifying feasibility of the approach. The same suppression for *appY* was achieved by three cycles, while eightfold transcription activation required nine iterations of mutagenesis. In both cases, the number of potential start points decreased resulting in an ordinary regulatory region with only one dominant promoter in the case of positive selection. Efficiency of H-NS binding remained virtually unchanged in all mutant constructs. Based on these findings we conclude that excessive promoters can adversely affect transcription by providing a platform for interference between several RNA polymerase molecules, which can act as a silencer at promoter-dense regions.

## Introduction

Horizontal gene transfer plays a pivotal role in bacterial evolution assisting in the adaptation of microbes to the environment and increasing the diversity of their populations (Gogarten et al., 2002; Wiedenbeck and Cohan, 2011; Cordero and Polz, 2014). At least five mechanisms allow bacteria to capture alien genetic material, including conjugation (Bañuelos-Vazquez et al., 2017; Delavat et al., 2017), transduction (Keen et al., 2017), transformation (Overballe-Petersen et al., 2013) and transport within either outer membrane vesicles (Tran and Boedicker, 2017) or phage-like particles (Lang et al., 2012; Grüll et al., 2018). Escaping bacterial defense systems, fragments of alien DNA with a certain probability can incorporate into the genome of a new host, where they can be identified based on the contextual difference from the rest of the nucleotide sequence (Lawrence and Ochman, 1998; Nakamura et al., 2004; Price et al., 2008; Langille and Brinkman, 2009; Huang et al., 2012). Although it is still unknown how bacteria integrate foreign genes into their regulatory networks, the recombinant areas turned out to be enriched with AT base pairs (Daubin and Ochman, 2004), and increased frequency of promoter-like sequences has been already regarded as a signature of foreign genes (Huang et al., 2012).

A typical regulatory region of bacterial genes contains one or several overlapping promoters with one or several transcription start points (TSPs) in each (Gama-Castro et al., 2016). However, there is a tendency to initiate transcription from a single site, which is sometimes regulated by superimposed promoters recognized by different σ-factors (Panyukov and Ozoline, 2013). Hence, it was surprising to find 78 “promoter islands” with extremely high density of potential TSPs, using a promoter finder PlatProm (Shavkunov et al., 2009). All these “islands” formed complexes with RNA polymerase and initiated synthesis of short oligonucleotides, whereas full-length transcription was barely detected (Panyukov and Ozoline, 2013; Panyukov et al., 2013). The biological expediency of such suppression became clear after it turned out that 75 out of 78 “islands” were associated with genes horizontally acquired by *Escherichia coli* (*E. coli*).

Most transferred genes are useless for the cell, and bacteria have elaborated mechanisms for their silencing. In *E. coli* this function is performed by a specific sentinel, a histone-like protein H-NS (Lucchini et al., 2006; Dorman, 2007), which inhibited transcription from all of the tested “promoter islands” (Purtov et al., 2014). Given that association with RNA polymerase is a general mode of transcription repression by H-NS (Oshima et al., 2006), excessive promoters may emerge near alien genes due to spontaneous mutagenesis evolutionarily aimed to create a platform for this combined binding. In this case, an increase in the transcriptional activity of the “islands” should be accompanied by a decrease in the number of potential binding sites for RNA polymerase and/or H-NS. Here we confirmed this hypothesis for RNA polymerase using error-prone PCR and a reporter plasmid for the selection of mutated genomic regions with increased and decreased promoter activity.

## Materials and Methods

### Bacterial Strains

Transcriptional activity of promoter mutants was estimated in *E. coli* Top 10 cells. Model DNA fragments were amplified from the genome of *E. coli* K12 MG1655 (GenBank NC_000913.3). Cells of *E. coli* BL21(DE3) or *E. coli* BL21(DE3)Δ*hns* transformed with pGEM_H-NS-His expression vector (Tutukina et al., 2015) were used to purify recombinant H-NS or to obtain cellular lysates enriched with H-NS-His.

### Plasmids and DNA Fragments

**FIGURE 1:**
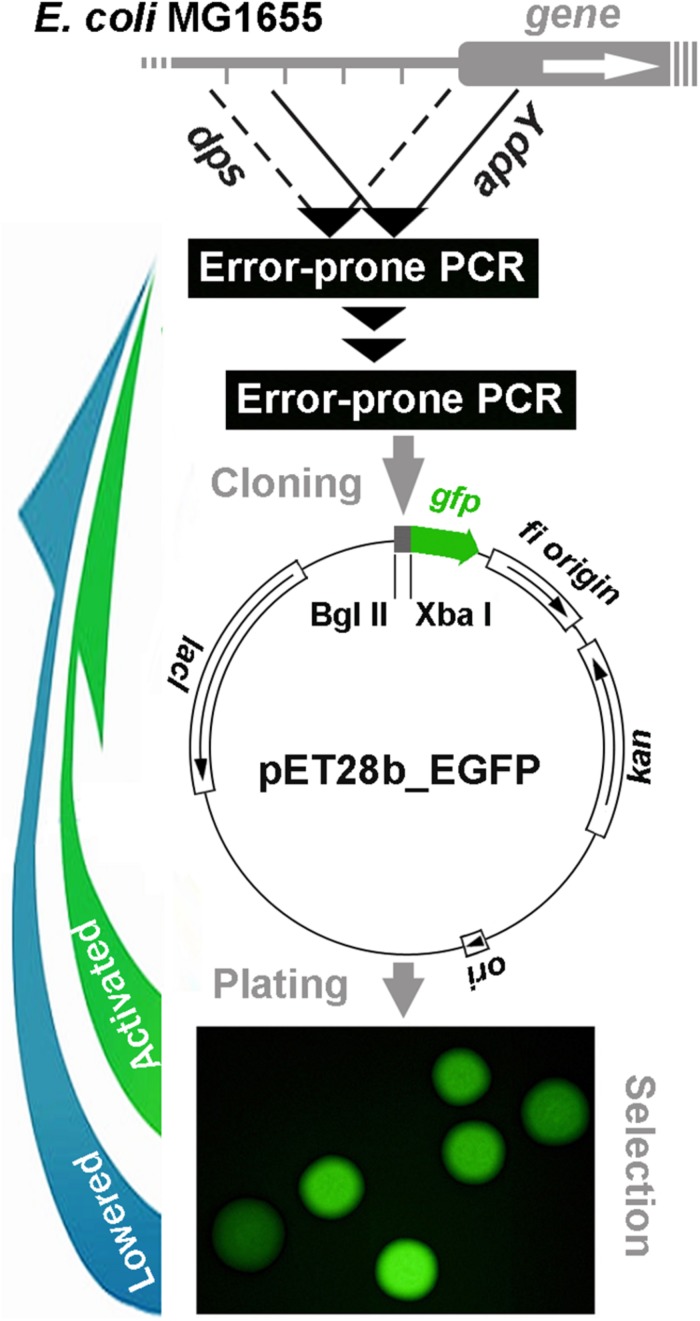
Schematic representation of cyclic error-prone mutagenesis for model regulatory regions with a reporter plasmid-mediated selection of mutant derivatives applied in the present study.

Plasmid pET28b-EGFP with the *gfp* gene encoding green fluorescent protein (Masulis et al., 2015) was used as a reporter vector (Figure 1). At the preliminary step, the fragments subjected to mutagenesis were amplified with Taq DNA polymerase (Evrogen, Russia) from the purified genomic DNA of *E. coli* MG1655 using Biometra T1 thermocycler (Germany). A fragment containing the regulatory region of the *dps* gene (402 bp) was obtained with primers: 5′-TCCTCTAGATGTTATGTCCCAGT-3′ and 5′-GGAAGATCTTCCTCGGAGAAACACT-3′ (underlined are restriction sites for *Xba*I and *Bgl*II, respectively). A fragment with the *appY*-associated “promoter island” (423 bp) was amplified with primers: 5′-GATAAGATCTGCAAGTAAAAATGATACTC-3′ and 5′-CCCTTCTAGATTTGTCGCTTACAATAAA-3′. All PCR reactions were carried out using a standard protocol: 2 min melting at 95°C followed by 35 cycles: 95°C, 30 s; 55°C, 30 s; 72°C, 1 min.

### Error-Prone PCR Mutagenesis

After purification of DNA samples with QIAquick PCR Purification Kit (Qiagen, Germany), error-prone PCR was carried out with GeneMorph II Random Mutagenesis Kit (Agilent, United States). Without purification, 0.1 ng of amplicon samples were used for a second stage of PCR. Thereafter, amplicons were purified with QIAquick PCR Purification Kit and prepared for cloning into pET28_EGFP vector using FastDigest *Xba*I and FastDigest *Bgl*II nucleases (Thermo Fisher Scientific, United States). Restriction was carried out in universal FastDigest buffer for 1 h at 37°C. Restriction fragments were purified with QIAquick PCR Purification Kit, while the pET28_EGFP vector processed in parallel was fractionated by gel electrophoresis in 1% agarose and purified with MinElute Gel Extraction Kit (Qiagen, Germany). Restriction fragments were ligated with T4 Ligase (Thermo Fisher Scientific, United States) immediately upstream of the *gfp* gene (12 h at 4°C) and the library of recombinant pET28b_eGFP (Figure 1) was used for transformation. Transformed cells were plated on 1.5% LB agar and incubated at 37°C overnight in the presence of kanamycin (80 μg/ml). GFP fluorescence was measured for individual colonies (exemplified in Figure 1) using Leica DM6000B fluorescent microscope (Germany) with excitation/emission at 480/510 nm and ImageJ software^1^. Several colonies with the highest and lowest level of fluorescence were selected and cultured for 12 h in LB broth, followed by isolation of plasmid with Plasmid Miniprep kit (Evrogen, Russia). Amplicons of target sequences were obtained with Taq polymerase (Evrogen, Russia) and sequenced. The mutated inserts with the largest number of new spontaneous mutations were used for a subsequent cycle of random mutagenesis, and the whole procedure was repeated until the GFP fluorescence was not distinguishable from the previous round.

### Fluorescence Measurements

The expression level of *gfp* in up-regulated constructs was compared to the *E. coli* cells transformed with corresponding pET28b-eGFP plasmids. For visualization, cells in LB medium (1 μl) freshly transformed with each of the constructed vectors were plated on one Petri dish and grown overnight before imaging. For quantitative measurements, cells were cultured for 12 h in 4 ml of LB medium, sedimented, suspended in 1 ml of TE buffer (pH 7.5) and sonicated (Misonix, United States). Cellular debris was sedimented and fluorescence intensity of GFP was measured in supernatant using a fluorescent spectrophotometer Cary Eclipse (Varian, Australia). The total protein concentration of the supernatant measured with a NanoDrop ND-1000 spectrophotometer (United States) was used for normalization.

### *In silico* Analysis

The distribution of potential TSPs was analyzed by a promoter finder PlatProm^2^ (Shavkunov et al., 2009). The binding sites for 109 transcription factors functioning in *E. coli* (listed in Supplementary Image 1) were searched by using RSAT full options matrix-scan software with *E. coli* K12 residue probabilities^3^ (Turatsinze et al., 2008). The position weight matrices for motifs recognized by 82 transcription factors were built using aligned sequences of their binding sites collected in RegPrecise^4^ (Novichkov et al., 2013). Matrices for 26 transcription factors, including H-NS, were taken from the Virtual Footprint collection^5^ (Münch et al., 2005), while the matrix for the nucleoid protein Dps was obtained using original ChIP-seq data (Antipov et al., 2017). Sequence Logos for Supplementary Image 1 were generated by WebLogo 3^6^ (Crooks et al., 2004).

### Primer Extension

The total RNA was isolated from bacterial cells transformed with pET28b-EGFP containing different inserts as described (Masulis et al., 2015). Ten micrograms of RNA and 2 pmol of a ^32^P labeled *gfp*-specific primer 5′-CTCTGGTCAGGCAGATACCTCTGGTCAG-3′ were used for the synthesis of cDNA by RevertAid Premium reverse transcriptase (Thermo Fisher Scientific, United States). Samples were treated with RNase A (Thermo Fisher Scientific; 10 U, 37°C, 30 min), precipitated with threefold volume of 96% ethanol and 0.3M sodium acetate and washed with 70% ethanol. The precipitate was dissolved in 5 μl of 98% formamide with 8 mM NaOH and 4 mM EDTA, fractionated in 8% polyacrylamide gel (PAAG) with 8M urea and radioautographed.

### SDS PAGE Electrophoresis

SDS electrophoresis was used to assess the purity of H-NS. Stacking gel contained 4% acrylamide, 0.125M Tris-HCl (pH 6.8), 0.1% SDS. Separating gel contained 12.5% acrylamide, 0.375 M Tris-HCl (pH 8.8), 0.1% SDS. Electrophoresis buffer included 25 mM Tris-HCl, 250 mM glycin and 0.1% SDS (pH 8.3). Ammonium persulfate and TEMED were added to a final concentration of 0.004 and 0.003%, respectively. Sample loading buffer contained 50 mM Tris-HCl (pH 6.8), 100 mM β-mercaptoethanol, 1% SDS, 0.01% bromphenol blue, and 10% glycerol. After sample loading the gels were run at constant current of 25 mA in stacking gel and 45 mA in separating gel. Molecular weight markers used were PageRuler Unstained Low Range Protein Ladder (Thermo Scientific, Lithuania) and Blue Prestained Protein Standard, Broad Range (NEB, United States). Following electrophoresis, the gels were stained in 0.25% Coumassie Brilliant Blue R-250 (Sigma, United States) with 45% ethanol and 10% acetic acid (Khimmed, Russia) and washed in solution containing 40% ethanol and 7% acetic acid.

### Electrophoretic Mobility Shift Assays (EMSA)

The original and mutagenized DNA fragments were amplified from isolated plasmids, purified, and used (0.5 pmol per reaction) to form complexes with H-NS under two different experimental settings. Complexes with purified H-NS were formed in 20 μl of buffer containing 5 mM Tris-HCl (pH 8.0), 1 mM MgCl_2_, 50 mM NaCl, 0.01 mM EDTA, 0.01 mM DTT and 3.3 mM imidazole (present in storage buffer of H-NS to prevent oligomerization) for 40 min at 30°C. Following the addition of glycerol to the final concentration of 10%, the samples were loaded on pre-warmed 5% PAAG. The gels were run at constant voltage of 280 V. Alternatively, H-NS protein was obtained from IPTG-induced (100 μM) *E. coli* BL21(DE3)Δ*hns* cells transformed with the pGEM_H-NS-His expression vector. Cells were harvested 4 h after the induction, washed, sonicated, and cellular debris was sedimented, while the supernatant containing 0.36–0.4 μg/μl of total protein was used for complex formation. Bands were stained with AgNO_3_ (pure protein) according to the protocol (Merril et al., 1979) or analyzed by Western blotting (cell lysates) using anti-His-tag antibodies (Cell Signaling Technology, United States). Cross-reactivity test with anti-His antibodies using a plasmid-less lysate of cells with deleted *hns* gene has been shown to be negative in a previous publication (Tutukina et al., 2015). The intensity of complexes formed by mutagenized derivatives was compared to that of native fragments using ImageJ. SigmaStat package of SigmaPlot was implemented for statistical analysis^7^.

## Results

### Selected Genomic Regions

**FIGURE 2:**
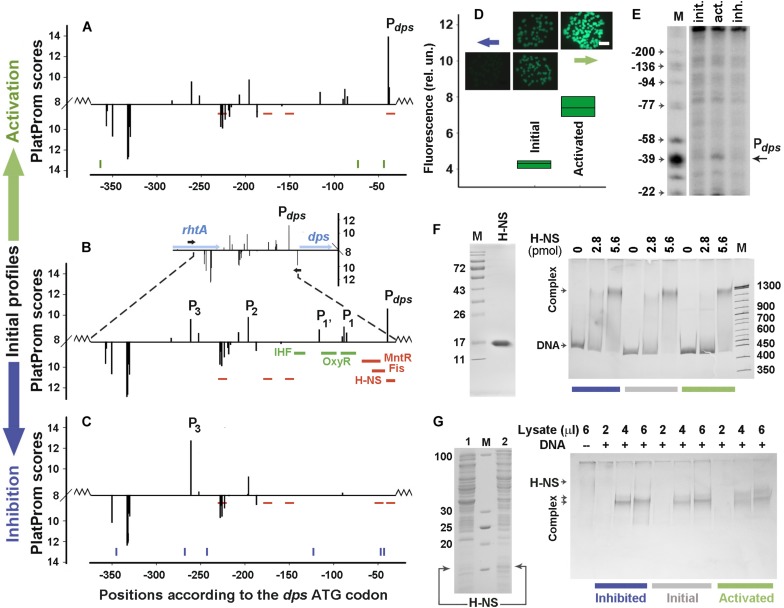
Error-prone PCR mutagenesis of the *dps* promoter region. **(A–C)** The distribution of TSPs (bars) predicted by PlatProm in the wild-type **(B)** and mutated **(A,C)** sequences (*p* < 0.00004). The vertical bars above and below from the X-axes mark TSPs of promoters predicted on the top and bottom strands, respectively. Their amplitudes correspond to the PlatProm scores. An insert in **(B)** shows the profile obtained in the genomic environment, while the main panel demonstrates the scores calculated for the plasmid insert (bordered by zigzag lines). Horizontal blue and black arrows show the positioning of genes and primers used for cloning, respectively. Thick colored lines indicate known binding sites for IHF, OxyR, MntR, Fis, and H-NS. Thin red lines show predicted sites for H-NS. Ticks in **(A,C)** show the locations of point mutations generated by random mutagenesis. **(D)** Mutation-mediated changes in the *gfp* expression visualized by fluorescent microscopy (colonies) and measured in lysates using a fluorescent spectrophotometer (box plots). The exposition time for imaging was 190 ms. Box plots represent five independent experiments. **(E)** Primer extension assays carried out with the *gfp*-specific primer. The total RNA was purified from the cells transformed with the initial reporter vector and mutant derivatives obtained after negative and positive selection. DNA markers (M) indicate positions in respect to the *dps* ATG codon. **(F)** The purity of H-NS and EMSA of purified protein with 0.5 pmol of DNA fragments amplified from the initial or mutagenized fragments. **(G)** SDS-PAGE with lysates obtained from *E. coli* BL21(DE3)Δ*hns* cells transformed with pGEM_H-NS-His before (line 1) or after (line 2) IPTG induction and EMSA carried out with the same DNA fragments as in **(F)**. The amount of the pure H-NS **(F)** and the volume of lysate **(G)** used are indicated above the gels. Protein concentration in the lysate was 0.36 μg/μl.

Two H-NS-dependent promoter regions were selected for error-prone mutagenesis. One of them belongs to the inherent *E. coli* gene encoding the nucleoid protein Dps (Figures 2A–C). It was selected to assess the capacity of random mutagenesis to affect the activity of normal promoters. Having no “promoter island,” this regulatory region contains the main promoter P*_*dps*_* (Altuvia et al., 1994), three weak promoters P_1_, P_1__’_, P_2_ and a distal promoter P_3_, which affects the strength of P*_*dps*_* (Shvyreva et al., 2011). Nucleoid proteins Fis and H-NS, as well as local repressor MntR are inhibitors of P*_*dps*_*, whereas OxyR and IHF activate this promoter upon oxidative stress and/or transition to stationary growth (Gama-Castro et al., 2016).

**FIGURE 3:**
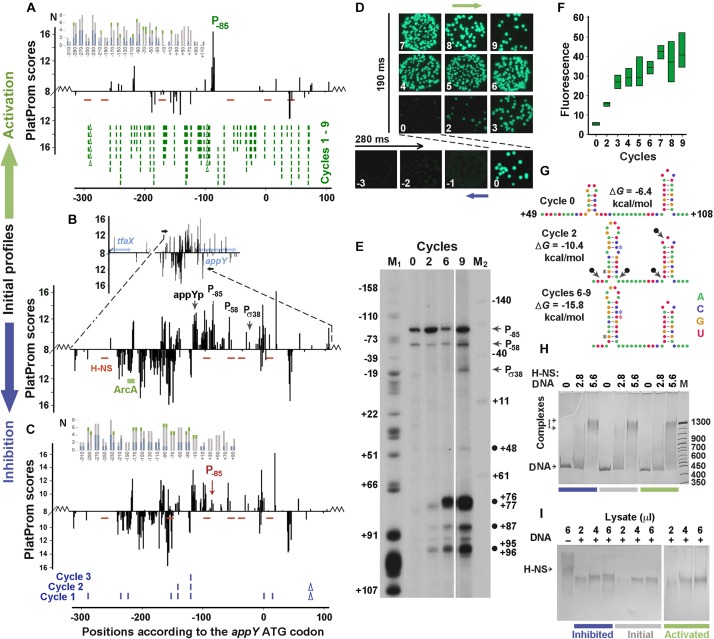
Error-prone PCR mutagenesis of the *appY* promoter region. **(A–C)** The distribution of TSPs (bars) predicted by PlatProm in the wild-type **(B)** and mutated **(A,C)** sequences (*p* < 0.00004) are plotted as described in Figure 2 legend. Green rectangle indicates known binding site for ArcA. Red lines mark the predicted sites for H-NS. Ticks and Δ symbols in **(A,C)** show the location of point substitutions and deletions, generated by random mutagenesis, respectively. **(D,F)** Mutation-mediated changes in the expression of *gfp* visualized in bacterial colonies by fluorescent microscopy **(D)** and measured in lysates (box-plots) by spectrofluorimetry **(F)**. Exposition times for positively and negatively selected samples are indicated. Box plots represent five independent experiments. **(E)** Primer extension assays carried out with the *gfp*-specific primer from the total RNA purified from the cells transformed with the initial reporter vector (cycle 0) and the mutant derivatives obtained at cycles 2, 6, and 9. DNA markers M_1_ (G-sequencing ladder) and M_2_ (50 bp ladder end-labeled with ^32^P using T4 polynucleotide kinase) indicate positions in respect to the initiation codon of *appY*. Black circles mark cDNA products, which can result from nuclease digestion. **(G)** Mutation-mediated changes in the folding propensity of RNA near the indicated positions. **(H,I)** EMSA carried out with 0.5 pmol DNA fragments amplified from the initial or mutagenized fragments with purified H-NS **(H)** or cellular lysates **(I)**. The amount of the pure H-NS **(F)** and the volume of lysate **(G)** used are indicated above the gels. Protein concentration in the lysate was 0.36 μg/μl.

The main model sample (Figures 3A–C) was taken from the “promoter island” associated with the *appY* gene, whose lateral transfer was justified by four different *in silico* approaches (Lawrence and Ochman, 1998; Nakamura et al., 2004; Langille and Brinkman, 2009; Huang et al., 2012). The gene *appY* encodes a transcription activator of at least two horizontally transferred operons – *appCBXA* and *hyaABCDEF* (Gama-Castro et al., 2016). The fragment contains 171 TSPs (Figure 3B) predicted on both strands by the promoter finder PlatProm (Shavkunov et al., 2009), but only those with TSPs located 114 bp (appYp) and 25 bp (P_σ__38_) upstream from the ATG codon were previously identified as functional by high-throughput techniques (Huerta and Collado-Vides, 2003; Maciag et al., 2011, respectively). The expression of the *appY* gene is inhibited by H-NS (Atlung et al., 1996; Purtov et al., 2014) and DpiA (CitB) (Ingmer et al., 1998) and can be activated by ArcA (Lynch and Lin, 1996). However, the binding site was experimentally identified only for ArcA (Lynch and Lin, 1996). Since PlatProm predicts TSPs, by taking into account the presence of promoter-specific motifs in a wide flanking area (from −250 to + 150 bp), the transfer of model regulatory regions into a plasmid slightly changed the profiles of potential TSPs at the borders of integration, which is shown in the main panels and inserts of Figures 2B, 3B. Due to the multiplicity of promoters in both model sequences, all positional coordinates are further referred in accordance with the ATG codons of the *dps* and *appY* genes. The sequences of both genomic regions are given in Supplementary Data Sheets 1, 2.

### Error-Prone Mutagenesis Affected the Strength of P_*dps*_

Only one round of negative or positive selection was sufficient to lower the transcriptional activity of the *dps* regulatory region to the background level or to activate the expression of the reporter gene (Figures 2A–D). Six mutations were obtained in the down-regulated derivative (blue ticks at the bottom of Figure 2C and sequences in Supplementary Data Sheet 1). One of them (position −47), which is 8 bp upstream of the P*_*dps*_* TSP, lowered its score from 10.6 to 6.6 by replacing a conservative T in the −10 element of this promoter with A (TATACT → TATACA, consensus: TATAAT). This also created a weak (*p* = 9.0 × 10^–4^) H-NS binding site in position −54 (Figure 2C). Another mutation (123 bp upstream of ATG) simultaneously disturbed the up-element of P_1_ and the −10 element of P_1’_ (TTTAGTTTT → TTTAGTTGT and TAGTTT → TAGTTG, respectively). Substitution A→T in position −268, on the contrary, improved the −10 element of P_3_ (TAACCA → TAACCT), but without a compensatory effect on the transcriptional activity reduced by the two aforementioned mutations. This independence of *gfp* expression from P_3_ in the plasmid pET28b_EGFP corresponds to our previous data, indicating that P_3_ and P_2_ are required for maximum transcription of *gfp*, but cannot ensure its expression in the absence of P_1’_, P_1_, and P_*dps*_ (Shvyreva et al., 2011).

Positive selection was attained by only three substitutions (green ticks in Figure 2A and sequences in Supplementary Data Sheet 1). One of them fell into the −35 element of P*_*dps*_* (position −73) and shifted its context closer to the consensus sequence (TAGCGG → TTGCGG, consensus: TTGACA). Primer extension assays confirmed the activation of P*_*dps*_* (Figure 2E). The other two mutations (at positions −44 and −364) did not affect specific modules in any promoter. Moreover, the substitution at −44 turned out to be the same (A→G) as in the suppressed construct (Figures 2A,C and Supplementary Data Sheet 1). Thus, the substitution in the −35 element of P*_*dps*_* is most likely responsible for a twofold increase in the *gfp* fluorescence (Figure 2D).

Since in the down-regulated derivative mutation at position −47 increased the conformity to the context of H-NS binding sites, we compared the H-NS affinity to both mutated constructs with that of the initial fragment by EMSA (Figures 2F,G). When pure H-NS protein (left panel in Figure 2F) was used, all fragments were retained in smeared complexes with electrophoretic mobility being highly dependent on the concentration of the protein. Densitometry analysis indicated that the binding efficiency of the activated mutant remained the same as that of the initial fragment (100.2 ± 4.1%, *n* = 6), while interaction with the down-regulated construct showed only statistically insignificant tendency for increase (117 ± 13.7%, *n* = 6). As promoter mutants were selected *in vivo* (Figure 1), certain contribution to the expression of *gfp* can be provided by some cellular agents absent *in vitro*. We therefore performed parallel EMSA experiments using the same DNA samples with lysates obtained from bacterial cells overproducing H-NS (left panel in Figure 2G). In this case, all fragments tend to retain the protein in two complexes (Figure 2G) indicating that cellular components indeed affect the H-NS binding to the model fragments, but the total amount of the protein bound to the transcriptionally inactive construct was again approximately the same as for the native fragment (112.8 ± 4.7%, *n* = 6). Surprisingly, the up-regulated derivative, whose substitutions did not change any of H-NS binding sites, reproducibly formed the largest complex with a higher efficiency than the two other fragments (Figure 2G) and demonstrated a slightly higher affinity to the protein (128.9 ± 9.1%, *p* = 0.537, *n* = 6). Assuming the slight difference between the smaller and larger complexes cannot be mediated by binding of a large protein, like RNA polymerase, we suggested that the mutation-mediated increase in binding may be due to some other transcription factors. Thus, we searched for potential binding sites of 109 regulatory proteins with RSAT matrix-scan software and found them for Dps at position −370/−363 (*p* = 4.7e-4) and AraC at position −94/−57 (*p* = 8.1e-4). Of these binding sites, the one with increased affinity for the AraC dimer (monomer MW – 33.4 kDa) may explain the observed change in the mobility of the complexes. Thus, it became clear that the functionality of the ordinary promoter region is highly sensitive to random mutagenesis and quite expectedly accumulated changes in the context of key promoter elements in response to positive or negative selection.

### Mutation-Mediated Activation of the *appY* Regulatory Region Eliminated Excessive Promoters

Both negative and positive selection reduced the number of potential promoters in the *appY*-associated “promoter island” (Figures 3A–C, sequences in Supplementary Data Sheet 2 and PowerPoint presentation in Supplementary Presentation 1). The expression of *gfp* was suppressed to the background level by one deletion and eight point substitutions obtained in three cycles of negative selection (Figures 3A,C,D). Many potential promoters stayed unaffected, but the score of the promoter with a maximum yield (primer extension assay in Figure 3E, cycle 0) decreased due to the point substitution in its −35 element TTGCAA → CTGCAA (TSP at position −85). Thus, just as for the *dps* regulatory region (Figure 2), a mutation affecting the most active promoter was obtained in the first cycle of mutagenesis, indicating the highest dependence of transcriptional activity on the context of the dominant promoter, even in a promoter-dense region.

Nine rounds of mutagenesis were required to activate this area to the maximal level (Figures 3D,F). This generated 49 substitutions with two deletions and turned the “promoter island” into an ordinary regulatory region possessing only one dominant promoter cluster (Figure 3A and PowerPoint Presentation 1 in Supplementary Materials, step by step demonstrating all successive changes). Predominantly selected among many other promoter-like sequences in the native construction (Figure 3E, cycle 0), this cluster remains active in all derivatives. At the end of the experiment, its score increased from 14.76 to 16.8 due to two point mutations and one deletion that changed the −10 element to a nearly consensus sequence (TAAAAAT → TATACT), while the scores of many other promoter-like sequences decreased. At the same time, two mutations, obtained in the first (T_+70_ → C) and the second (A_+54_ → G) cycles, extended the hairpin originally formed in the region of + 60/ + 71 to + 53/ + 76, and the substitution C_+71_ → T ensured the perfect complementarity in this hairpin (Figure 3G and Supplementary Data Sheet 2). Being subjected to cleavage by cellular stem–loop – specific endonucleases (for instance RNase E or RNase III), such structures may give products marked by black circles in Figure 3E (near positions + 48, + 87 and + 95/96). In addition, early termination of reverse transcription at the 3′-ends of the large hairpin can explain the appearance of the main product initiated from the plasmid-specific primer and terminated at positions + 76/ + 77. Since there were no substitutions in the first two cycles that simultaneously increased the scores of the three promoters (TSPs at positions + 76/ + 77, + 87, and + 95/96), it seems unlikely that these bands correspond to new transcription units. Therefore, the intensity of these bands may reflect an increase in the transcriptional output of the entire recombinant region, which roughly corresponds to the eightfold enhanced yield of GFP accumulated in the cells (Figures 3D,F).

As a foreign gene, *appY* is strongly inhibited by H-NS (Atlung et al., 1996; Purtov et al., 2014). Since the binding sites for this protein have not been identified experimentally, we searched them *in silico* and revealed five potential contact regions (red dashes in Figure 3A), although with a rather low reliability (1.1e-3 ≤ *p* ≤ 5.0e-3). Error-prone mutagenesis created one new site at position −160 in the down-regulated mutant (Figure 3C, *p* = 3.8e-3) that can contribute to the slightly increased affinity of H-NS to this construct: 110.1 ± 5.7 and 117.2 ± 0.8% in the EMSA experiments with pure protein and lysates (Figures 3H,I, respectively) these differences are not statistically significant (*p* = 0.78 and 0.15, respectively, n = 6). Multiple mutations in the activated construct created three additional sites (positions −296, −173 and + 41, 2.1e-3 ≤ *p* ≤ 3.8e-3) eliminating two binding modules at positions −102 and −45 (Figure 3A). Although their number turned out to be higher than in the control sample, the affinity of the mutant derivative stayed unchanged: 102.4 ± 6.1 and 97.2 ± 5.3% (*n* = 6) in assays with pure protein and lysates (Figures 3H,I, respectively). Thus, it became clear that the selection of derivatives with increased activity does not lead to the selection of mutants with a reduced affinity for H-NS.

To test the possibility that blindly selected mutations in the up-regulated derivative predominantly accumulated new motifs for activators or eliminated binding sites for inhibitors and gave the opposite effect for an inhibited construct, we grouped 109 transcription factors with known binding motifs into four functional categories (Supplementary Image 1). These include regulators known to act only as inhibitors (“inh”), only as activators (“act”), functionally dependent from promoter or ligand (“d”) and regulatory proteins with an uncharacterized mode of action (“?”). Using RSAT with *p* ≤ 1e-3 as a threshold, we found 95 binding sites for 50 transcription factors in the initial sequence. Of these, 53 were targets for double regulators, 29 for known inhibitors, and only 7 sites for activators. Nine mutations of the down-regulated derivative left this proportion (54, 27, and 7, respectively) and the total number of transcription factor binding motives (96) almost the same as shown in Figure 3C. Multiple mutations of up-regulated construct reduced its AT-content from 74.9 to 69.1%. As a consequence, the total number of predicted binding sites decreased to 81, but the proportion of sites for regulators with different effects (45, 24, and 7, respectively), as well as their distribution along the recombinant area (insert in Figure 3A) again did not show any specific bias. Thus, we conclude, that the selection of mutants with altered expression of the reporter gene is primarily mediated by the selection of the derivative with a suppressed main promoter in the down-regulated mutant and the derivative without competing promoters in the activated mutant.

## Discussion

Exploiting foreign genes for rapid environmental adaptation, bacteria also have to evolve the mechanisms for their acquisition or suppression. We hypothesized that recombination increases local mutagenesis with predominant accumulation of AT base pairs. This can be achieved by deamination of cytidines or oxidation of guanines (Panyukov et al., 2013; Kiselev et al., 2017), if nucleotide sequences in the recombinant area are more sensitive for such modifications. As a result, AT-rich H-NS binding sites capable of suppressing transcription and promoter-like regions suitable for controlling expression of beneficial genes can be evolved. Enhanced mutagenesis in the recombinant area and an assumption that excessive RNA polymerase binding sites can negatively affect transcription, are the two key points in our hypothesis. To test the first assumption, we transferred two foreign genes into the *E. coli* MG1655 genome and started a long-term evolutionary experiment that already confirmed an increased frequency of spontaneous GC to AT substitutions in the recombinant region (Glazunova et al., 2016). Here, we confirmed the second assumption by obtaining an up-regulated *appY* mutant with dramatically decreased number of potential binding sites for RNA polymerase.

The idea of the bacterial RNA polymerase to be a repressor of transcription was implicitly or directly announced before. Though transcription inhibition, as opposed to the main synthetic role, is not regarded as its general function, there are a number of studies indicating that the enzyme complex formation with DNA can suppress transcription from neighboring promoters (Nieuwkoop and Bender, 1988; Masulis et al., 2015). For instance, in the promoter region of the hutUH operon investigated in the genome of *Klebsiella aerogenes* (Nieuwkoop and Bender, 1988), the binding of RNA polymerase to a mutated P_UH_ promoter with a strengthened −10 region was shown to block the transcription from the overlapping weaker P_C_ site. Another way of transcription blockade is related to the inactivation of a downstream promoter under active functioning of an upstream promoter. This phenomenon can be exemplified by intragenic promoters required for the synthesis of an RNA product from within the gene of 16S rRNA and activated in the stationary phase upon repression of transcription of the major promoters of the *rrn* operon (Takada et al., 2016). A total of 2,701 sites for binding of RpoD holoenzyme were identified (Shimada et al., 2014) by Genomic SELEX screening. Of these, 1626 promoters turned out to be located inside 777 open reading frames, thus implying a potential functional “conflict” between the promoters located outside of coding regions and adjacent to them intragenic sites of interaction, and there are numerous examples of RNA polymerase complex formation with intragenic promoters (Tutukina et al., 2007; Masulis et al., 2019).

Starting these experiments with promoter regions regulated by H-NS, we expected to observe changes in the number of its potential binding sites, types of complexes formed or efficiency of interaction. However, only the activated construct with the *dps* regulatory region demonstrated some mutation-driven alterations in complex formation (Figure 2G). Thus, we conclude that the decrease in the number of promoter-like sites is the main driving force for the enhanced transcriptional activity in the *appY* construct. If the population control over horizontally transferred genes primarily operates at the level of randomly created promoters, then a new function of RNA polymerase as a sentinel of foreign genes should be considered.

## Data Availability Statement

All datasets generated for this study are included in the article/Supplementary Material.

## Author Contributions

AB and OG: isolation of plasmids for sequencing. AB: PCR mutagenesis and GFP fluorescence measurements for cell extracts. OA and KS: primer extension analysis. OA: electrophoretic mobility shift assay. NS and H-NS: isolation/purification. IM: *gfp*-encoding reporter plasmid. KS: initial plasmid construct. OO: bioinformatic analysis and manuscript preparation for publishing with a contribution of all other authors.

## Conflict of Interest

The authors declare that the research was conducted in the absence of any commercial or financial relationships that could be construed as a potential conflict of interest.

## Footnotes

1 https://imagej.net/Welcome

2 http://www.mathcell.ru/model6.php?l=en

3 http://embnet.ccg.unam.mx/rsat/matrix-scan-quick_form.cgi

4 http://regprecise.lbl.gov/RegPrecise

5 http://www.prodoric.de/vfp/vfp_promoter.php

6 http://weblogo.threeplusone.com/create.cgi

7 http://www.sigmaplot.co.uk/products/sigmaplot/statistics.php
