# Supplementary figures and images for: Excessive Promoters as Silencers of Genes Horizontally Acquired by Escherichia coli
Source: Front Mol Biosci. 2020 Feb 26;7:28. doi: 10.3389/fmolb.2020.00028 (PMC7054387; doi:10.3389/fmolb.2020.00028)

## Slide 1
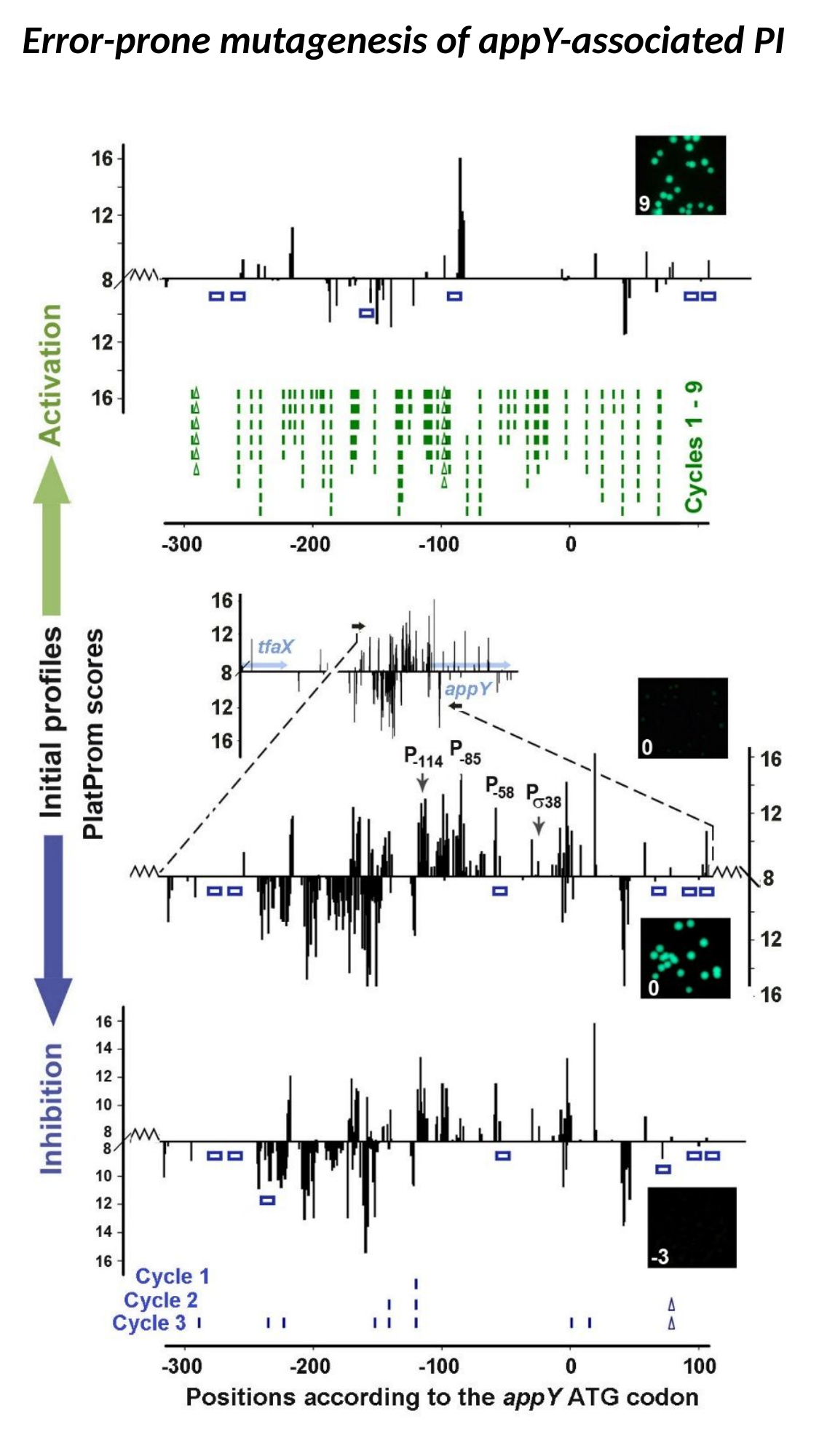

Error-prone mutagenesis of appY-associated PI

Supplement: Supplementary file 1 [file Presentation_1.PPTX]

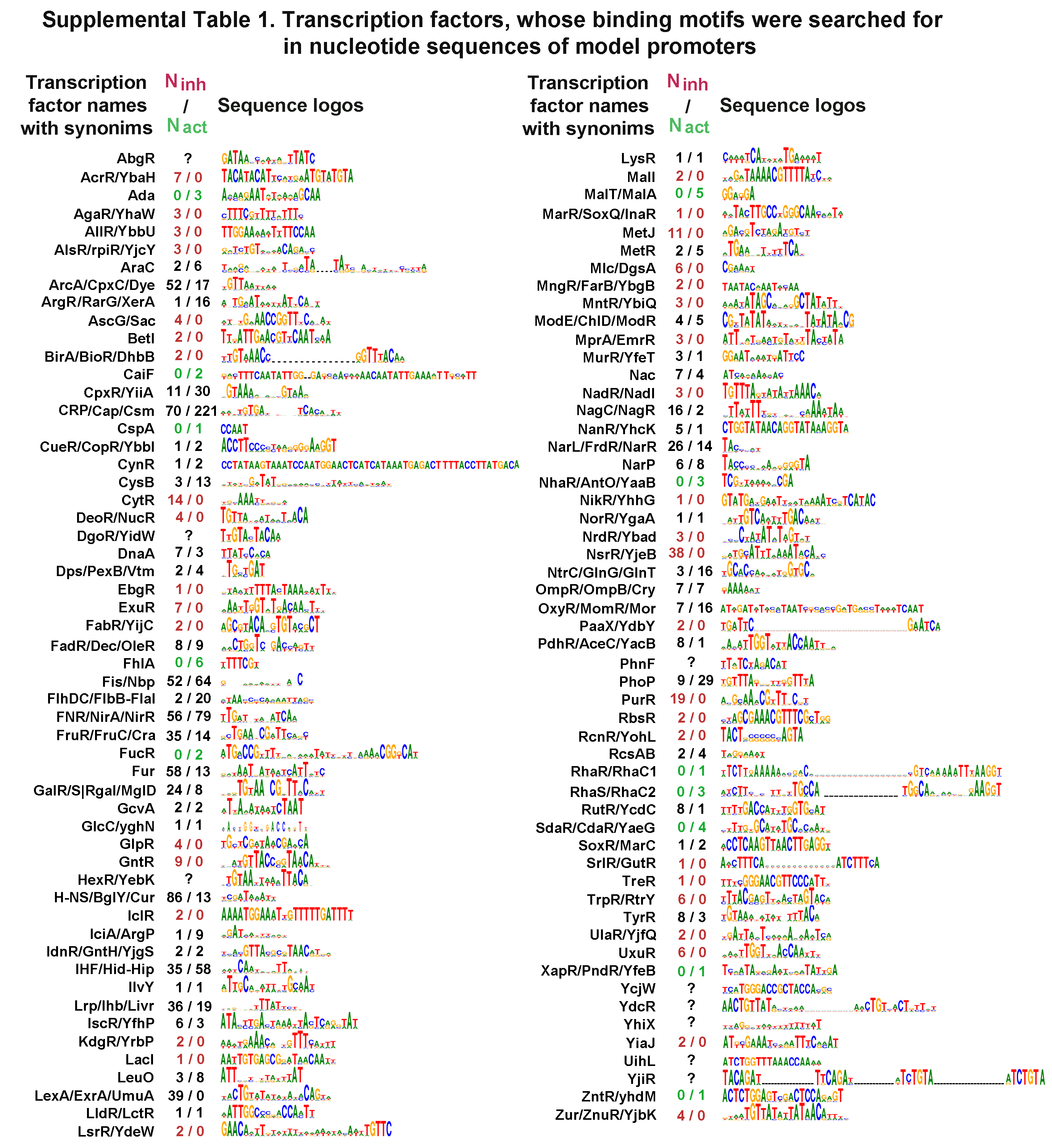

Supplement: Supplementary file 2 [file Image_1.JPEG]
