# Supplementary material for: Excessive Promoters as Silencers of Genes Horizontally Acquired by Escherichia coli
Source: Front Mol Biosci. 2020 Feb 26;7:28. doi: 10.3389/fmolb.2020.00028 (PMC7054387; doi:10.3389/fmolb.2020.00028)
Supplement: Supplementary file 3 [file Data_Sheet_1.PDF]

Sequences of model fragment taken from the *dps* promoter region and mutagenized constructs

Substitutions are marked by red and blue in upregulated and downregulated mutants, respectively.
Linker sequences are bolded.
The ATG codon of *dps*, P*dps* P1, P1’ P2 and P3 are bolded and underlined.
TSPs of P*dps* P1, P1’ P2 and P3 are located in positions -39, -85, -116, -207/-196 and -261, respectively.

Mutagenized sequence of the *dps* promoter region

AGATCTTCCTCGGAGAAACACTGACACCCATACAGCTACTGGCGCTCGGCGCTATCATCGCCGCTTCAATGGGGTCT
ACGCTGACAGTACGCAAAGAGAGCAAAATAAAAGAATTAGACATTAATTAAATTTACATTTCTGCATGGTTATGCAT
AACCATGCAGA**A**TTTCTCGCTACTTTTCCTCTACACCGTCTTTATATATCGAATTATGCAAAAGC**A**TATTTATTCC**G**
AAAATTCCTGGCGAGCAGATAAATAAGAATTGTTCTTATCAATATATCTAACTCATTGAATCTTTATTAGTTTTGTT
TT**T**CACGCTTGTTACCACTATTAGTGTGATAGG**A**ACAGCCAGAATAGCGGAACACATAGCCGGTGCTATACTTAATC
TC**G**TTAATTACTGGGACATAACA**TCTAGA**

Sequence of the *dps* promoter region prolonged to the ATG codon

AGATCTTCCTCGGAGAAACACTGACACCCATACAGCTACTGGCGCTCGGCGCTATCATCGCCGCTTCAATGGGGTCT
ACGCTGACAGTACGCAAAGAGAGCAAAATAAAAGAATTAGACATTAATTAAATTTACATTTCTGCATGGTTATGCAT
AACCATGCAGA**A**TTTCTCGCTACTTTTCCTCTACACCGTCTTTATATATCGAATTATGCAAAAGC**A**TATTTATTCC**G**
AAAATTCCTGGCGAGCAGATAAATAAGAATTGTTCTTATCAATATATCTAACTCATTGAATCTTTATTAGTTTTGTT
TT**T**CACGCTTGTTACCACTATTAGTGTGATAGG**A**ACAGCCAGAATAGCGGAACACATAGCCGGTGCTATACTTAATC
TC**G**TTAATTACTGGGACATAACATCAAGAGGATATGAAATT**ATG**

Positive selection for the *dps* promoter region

AGATCTTCCTCGGAGAAACACTGACACCCATACAGCTACTGGCGCTCGGCGCTATCATCGCCACTTCAATGGGGTCT
ACGCTGACAGTACGCAAAGAGAGCAAAATAAAAGAATTAGACATTAATTAAATTTACATTTCTGCATGGTTATGCAT
AACCATGCAGA**A**TTTCTCGCTACTTTTCCTCTACACCGTCTTTATATATCGAATTATGCAAAAGC**A**TATTTATTCC**G**
AAAATTCCTGGCGAGCAGATAAATAAGAATTGTTCTTATCAATATATCTAACTCATTGAATCTTTATTAGTTTTGTT
TT**T**CACGCTTGTTACCACTATTAGTGTGATAGG**A**ACAGCCAGAATTGCGGAACACATAGCCGGTGCTATACTTAGTC
TC**G**TTAATTACTGGGACATAACATCAAGAGGATATGAAATT**ATG**

Negative selection for the *dps* promoter region

AGATCTTCCTCGGAGAAACACTGACACCCATACAGCTACTGGCGCTCGGCGCTATCATCGCCGCTTCAATGGGGTCT
ACGCAGACAGTACGCAAAGAGAGCAAAATAAAAGAATTAGACATTAATTAAATTTACATTTCTGCATGGTTATGCAT
AACCTTGCAGA**A**TTTCTCGCTACTTTTCCCCTACACCGTCTTTATATATCGAATTATGCAAAAGC**A**TATTTATTCC**G**
AAAATTCCTGGCGAGCAGATAAATAAGAATTGTTCTTATCAATATATCTAACTCATTGAATCTTTATTAGTTGTGTT
TT**T**CACGCTTGTTACCACTATTAGTGTGATAGG**A**ACAGCCAGAATAGCGGAACACATAGCCGGTGCTATACATAGTC
TC**G**TTAATTACTGGGACATAACATCAAGAGGATATGAAATT**ATG**

Changed promoters:

Pdps

ACCACTATTAGTGTGATAGGAACAGCCAGAATAGCGGAACACATAGCCGGTGCTATACTTAATCTC**g**TTAATTACTG
ACCACTATTAGTGTGATAGGAACAGCCAGAATTGCGGAACACATAGCCGGTGCTATACTTAGTCTC**g**TTAATTACTG
ACCACTATTAGTGTGATAGGAACAGCCAGAATAGCGGAACACATAGCCGGTGCTATACATAGTCTC**g**TTAATTACTG
-35 extended -10

P1

TATCTAACTCATTGAATCTTTATTAGTTTTGTTTTTCACGCTTGTTACCACTATTAGTGTGATAGG**a**ACAGCCAGAA
TATCTAACTCATTGAATCTTTATTAGTTGTGTTTTTCACGCTTGTTACCACTATTAGTGTGATAGG**a**ACAGCCAGAA
UP-element -35 -10

P1′

AGCAGATAAATAAGAATTGTTCTTATCAATATATCTAACTCATTGAATCTTTATTAGTTTTGTTTT**t**CACGCTTGTT
AGCAGATAAATAAGAATTGTTCTTATCAATATATCTAACTCATTGAATCTTTATTAGTTGTGTTTT**t**CACGCTTGTT
-35 -10

P3

GCAAAATAAAAGAATTAGACATTAATTAAATTTACATTTCTGCATGGTTATGCATAACCATGCAGA**a**TTTCTCGCTA
GCAAAATAAAAGAATTAGACATTAATTAAATTTACATTTCTGCATGGTTATGCATAACCTTGCAGA**a**TTTCTCGCTA
-35 -10
