# Supplementary material for: Excessive Promoters as Silencers of Genes Horizontally Acquired by Escherichia coli
Source: Front Mol Biosci. 2020 Feb 26;7:28. doi: 10.3389/fmolb.2020.00028 (PMC7054387; doi:10.3389/fmolb.2020.00028)
Supplement: Supplementary file 4 [file Data_Sheet_2.PDF]

Sequences of model fragment taken from the *appY* promoter island and mutagenized constructs obtained in 9 rounds of positive and 3 cycles of negative selection

All substitutions are marked by red.
A reverse mutation in cycle 7 is marked by green.
Deletions are indicated by “d” and colored blue.
Linker sequences are bolded.
The ATG codon of *appY* and the TSPs of promoters appYp, Pσ^{38} and P_{-85} are bolded and underlined.
Gray arrows show inverted repeats in the region +49 – +104

Wild type sequence of the *appY* model fragment

```
AGATCTGCAAGTAAAAATGATACTCTTTTTATTTTAAATTCAAACGGTTGACATATATATAGCAAGAGGTTTCAGGT
GCGTTGTAGTGAGTTTATGTTAATAAAAAGCATAGTAAGCGTTGAAAAATGTAACTTTGAAATAAGTTAGAATAAAA
AACAACATACATATAATAATTTAATCTTAAATGAAATTTATTAAAATTTGCAAACTATAATTTTGTGTATAAAAATA
TAAATGCACATCATCCTGATTATGATTGTGTATTTAATTGGTTGTTATTTGACTACTATCAACTTGTTTTAATTTTA
TGATAGGTGCAAGATGGATTATGTTTGCTCCGTAGTTTTCATCTGTCAATCATTTGATTTAATTATAAACAGGAGAG
TTATCTCGTTCAAAAAAAATTCATTGTTTATTGTAAGCGACAAATTCTAGA
```

Positive selection_cycle 1

```
AGATCTGCAAGTAAAAATGATACTCTTTTTATTTTAAATTCAAACGGTTGACATATATATAGCAAGAGGTTTCAGGT
GCGTAGTAGTGAGTTTATGTTAATAAAAAGCATAGTAAGCGTTGAAAAATGTAACTTTGTAATAAGTTAGAATAAAA
AACAACATACATATAATAATTTAATCTTAAATGAAGTTTATTAAAATTTGCAAACTATAATTTTGTGTATAAAAATA
TAAATGCACATTATCCTGATTGTGATTGTGTATTTAATTGGTTGTTATTTGACTACTATCAACTTGTTTTAATTTTA
TGATAGGTGCAAGATGGATTATGTTTGCTCCGTAGTTTTCATCTGTCAATCATTAGATTTAATTATAAACAGGAGAG
TTATCCCGTTCAAAAAAAATTCATTGTTTATTGTAAGCGACAAATTCTAGA
```

Positive selection_cycle 2

```
AGATCTGCAAGTAAAAATGATACTCTTTTTATTTTAAATTCAAACGGTTGACATATATATAGCAAGAGGTTTCAGGT
GCGTAGTAGTGAGTTTATGTTAATAAAAAGCATAGTAAGCGTTGAAAAATGTAACTTTGTAATAAGTTAGAATAAAA
AACAACATACATATAATAATTTAATCTTAAATGAAGTCTATTAAAATTTGCAAACTATAATTTTGTGTATAAAAATA
TAAATGCACATTATCCTGATTGTGATTGTGTATTTAATTGGTTGTTATTTGACTACTATCAACTTGTTTTAATTTTA
TGATAGGTGCAAGATGGATTATGTTTGCTCCGTAGTTTCCATCTGTCAATCATTAGATTTAATTATGAACAGGAGAG
TTATCCCGTTCAAAAAAAATTCATTGTTTATTGTAAGCGACAAATTCTAGA
```

Positive selection_cycle 3

```
AGATCTGCAAGTAAAAATGATACTCTTTTTATTTTAAATTCAAACGGTTGACATATATATAGCACGAGGTTTCAGGT
GCGTAGTAGTGAGTTTATGTTAATAAAAAGCATAGTATGCGTTGAAAAATGTAGCTTTGTAATAAGTTAGAATAAAA
AACAACATACATATAATAATTTAATCTTAAATGAAGTCTATTAAAATTTGCAAACTATAATTTTGTGTATdAAAATA
TAAATGCACATTATCCTGATTGTGATTGTGTATTTAATTGGTTGTTATTTGACTACTACCAACTTGTTTTAATTTTA
TGATAGGTGCAAGATGGATTATGTTTGCTCCGTAGTTTCCATCTGTCAATCATTAGATTTAATTATGAACAGGAGAG
TTATCCCGTTCAAAAAAAATTCATTGTTTATTGTAAGCGACAAATTCTAGA
```

Positive selection_cycle 4

```
AGATCTGCAAGTAAAAATGATACTCTTTTTAdTTTAAATTCAAACGGTTGACATATATATAGCACGAGGTTTCAGGT
GCGTAGTAGTGAGTTTATGTTAATAAAAAGCATAGTATGCGTTGAAAAATGTAGCTTTGTAATAAGTTAGAATAAGA
AACAACATACATATAACAATTTAATCTTAAATGAAGTCTATTAAAATTTGCAAACTATAAATTTGTGTATdAAACTA
TAAATGCACATTATCCTGATTGTGATTGTGTATTTAATTGGTTGTTATTTGACTACTACCAACTTGGTTTAATTTTA
TGATAGGTGCAAGATGGATTATGTTTACTCCGTAGTTTCCATCTGTCAATCATTAGATTTAATTATGAACAGGAGAG
TTATCCCGTTCAAAAAAAATTCATTGTTTATTGTAAGCGACAAATTCTAGA
```

Positive selection_cycle 5

```
AGATCTGCAAGTAAAAATGATACTCTTTATAdTTTAAATTCAAACGGTTGACATATATATAGCACGAGGTTTCATGT
GCGTAGTAGTGAGTTTATGTTATTAAAAAGCATAGTATGCGTTGAAAAATGTAGCTTTGTAATAAGTTAGAATAAGA
CTCAACATACATATAACAATTTAATCTTAAATGAAGTCTATTAAAATTTGCAAACTAACAATTTGCGTATdATACTA
TAAATGCACATTATCCTGATTGTGATTGTGTATTTAATTGGTTGTTATTTGACTACTACCAACTAGGTTTAATCTTA
TGATAGGTGCAAGATGGATTATGTTTACTCCGTAGTTTCCATCTGTCAATCATTAGATTTAATTATGAACAGGAGAG
TTATCCCGTTCAAAAAAAATTCATTGTTTATTGTAAGCGACAAATTCTAGA
```

Positive selection_cycle 6

AGATCTGCAAGTAAAAATGATACTCTTTATAdTTTAAATTCAAACGGTTGACATATATATAGCACGAGGTTTCATGT
GCGTAGTAGTGAGTTTATGTTATTAAAAAGCTTAGTATGCGTTGAAAAATGTAGCTTTGTAATAAGTTAGAATAAGA
CTCAACATACATATAACAATTTAATCTTAAATGAAGTCTATTAGAATTTGCAAACAAACAATTTGCGTATdATACTA
TAAATGCACATTATCCTGATTGTGATTGTGTATTTAACTGGTTCTTATTTGACTACTACCAACTAGGTTTAGTCTTA
TGATAGGTGCAGGATGGATTATGTTTACTCCGTAGTTTCCATCTGTCAATCATTAGATTTAATTATGAACAGGAGAG
TTATCCTGTTCAAAAAAAATTCATTGTTTATTGTAAGCGACAAA**TCTAGA**

Positive selection_cycle 7

AGATCTGCAAGTAAAAATGATACTCTTTATAdTTTAAATTCAAACGGTTGACATATATATAGCACGAGGTTTCATGT
GCGTAGTAGTGAGTTTATGTTATTAAACAGCTTAGTATGCGTTGAAAAATGTAGCTTTGTAATAAGTTAGAATAAGA
CGCGACATACATATAACAATTTAATCTTAAATGTAGTCTATTAGAATTTGCAAACAAACAATTTGCGTATdATACTA
TAAATGCACATCATCCTGATTGTGATTGTGTATTTAACTGGTTCTTATATGACTACTGCCAACTAGGTTTAGTCTTA
TGATAGGTGCAGGATGGATTATGTTTACTCCGTAGTTTCCATCTGTCAATCATTAGATTTAATTATGAACAGGAGAG
TTATCCTGTTCAAAAAAAATTCATTGTTTATTGTAAGCGACAAA**TCTAGA**

Positive selection_cycle 8

AGATCTGCAAGTAAAAATGATACTCTTTATAdTTTAAATTCAAACGGTTGACATATATATAGCACGAGGTTTCATGT
GCGTAGTAGTGAGTTTATGTTATTAAACAGCTTAGTATGCGTTGGAAAATGAAGCTTTGTAATAAGTTAGAATAAGA
CGCGACATACATATAACAATTTAATCTTAAATGTAGTCTATTAGCATTTGCAAACAAACAATTTGCGTATdATACTA
TAAATGCACATCATCCTGATTGTGATTGTGTATTTAACTGGTTCTTATATGACTACTGCCAACTAGGTTTAGTCTTA
TGATAGGTGCAGGATGGATTATGTTTACTCCGTAGTTTCCATCTGTCTATCATTAGATTTAATTATGAACAGGAGAG
TTATCCTGTTCAAAAAAAATTCATTGTTTATTGTAAGCGACAAA**TCTAGA**

Positive selection_cycle 9

AGATCTGCAAGTAAAAATGATACTCTTTATAdTTTAAATTCAAACGGTTGACATATATATAGCACGAGGTTTCATGT
GCGTAGTAGTGAGTTTATGTTATTAAACAGCTTAGTATGCGTTGGAAAGTGAAGCTTTGTAATAAGTTAGAATAAGA
CGCGACATACATATAACAATTTAATCTTAAATGTAGTCTATTAGCATTTGCAAACAAACAATTTGCGTATdATACTA
TAAATGCACATCATCCTGATTGTGATTGTGTATTTAACTGGTTCTTATATGACTACTACCAACTAGGTTTAGTCTTA
TGATAGGTGCAGGATGGATTATGTTTACTCCGTAGTTTCCATCTGTCTATCATTAGATTTAATTATGAACAGGAGAG
TTATCCTGTTCAAAAAAAATTCATTGTTTATTGTAAGCGACAAA**TCTAGA**

Wild type sequence of *appY* model fragment

AGATCTGCAAGTAAAAATGATACTCTTTTTATTTTAAATTCAAACGGTTGACATATATATAGCAAGAGGTTTCAGGT
GCGTTGTAGTGAGTTTATGTTAATAAAAAGCATAGTAAGCGTTGAAAAATGTAACTTTGAAATAAGTTAGAATAAAA
AACAACATACATATAATAATTTAATCTTAAATGAAATTTATTAAAATTTGCAAACTATAATTTTGTGTATAAAAATA
TAAATGCACATCATCCTGATTATGATTGTGTATTTAATTGGTTGTTATTTGACTACTATCAACTTGTTTTAATTTTA
TGATAGGTGCAAG**ATG**GATTATGTTTGCTCCGTAGTTTTCATCTGTCAATCATTTGATTTAATTATAAACAGGAGAG
TTATCTCGTTCAAAAAAAATTCATTGTTTATTGTAAGCGACAAA**TCTAGA**

Negative selection_cycle 1

AGATCTGCAAGTAAAAATGATACTCTTTTTATTTTAAATTCAAACGGTTGACATATATATAGCAAGAGGTTTCAGGT
GCGTTGTAGTGAGTTTATGTTAATAAAAAGCATAGTAAGCGTTGAAAAATGTAACTTTGAAATAAGTTAGAATAAAA
AACAACATACATATAATAATTTAATCTTAAATGAAATTTATTAAAATCTGCAAACTATAATTTTGTGTATAAAAATA
TAAATGCACATCATCCTGATTATGATTGTGTATTTAATTGGTTGTTATTTGACTACTATCAACTTGTTTTAATTTTA
TGATAGGTGCAAG**ATG**GATTATGTTTGCTCCGTAGTTTTCATCTGTCAATCATTTGATTTAATTATAAACAGGAGAG
TTATCTCGTTCAAAAAAAATTCATTGTTTATTGTAAGCGACAAA**TCTAGA**

Negative selection_cycle 2

AGATCTGCAAGTAAAAATGATACTCTTTTTATTTTAAATTCAAACGGTTGACATATATATAGCAAGAGGTTTCAGGT
GCGTTGTAGTGAGTTTATGTTAATAAAAAGCATAGTAAGCGTTGAAAAATGTAACTTTGAAATAAGTTAGAATAAAA
AACAACATACATATATTAATTTAATCCTAAATGAAATTTATTAAAATCTGCAAACTATAATTTTGTGTATAAAAATA
TAAATGCACATCATCCTGATTATGATTGTGTATTTAATTGGTTGTTATTTGACTACTATCAACTTGTTTTAATTTTA
TGATAGGTGCAAG**ATG**GATTATGTTTGCTCCGTAGTTTTCATCTGTCAATCATTTGATTTAATTATAAACAGGAGAG
TTATCTCGTTCdAAAAAAATTCATTGTTTATTGTAAGCGACAAA**TCTAGA**

Negative selection_cycle 3

AGATCTGCAAGTAAAAATGATACTCTTTTTATATTAAATTCAAACGGTTGACATATATATAGCAAGAGGTTTCAGGT
GCGTTGTAGCGAGTTTATGTTTATAAAAAGCATAGTAAGCGTTGAAAAATGTAACTTTGAAATAAGTTAGAATAAAA
AACAACATACATATATTAATTTAATCCTAAATGAAATTTATTAAAATCTGCAAACTATAATTTTGTGTATAAAAATA
TAAATGCACATCATCCTGATTATGATTGTGTATTTAATTGGTTGTTATTTGACTACTATCAACTTGTTTTAATTTTA
TGATAGGTGCAAG**GTG**GATTATGTTTGATCCGTAGTTTTCATCTGTCAATCATTTGATTTAATTATAAACAGGAGAG
TTATCTCGTTCdAAAAAAATTCATTGTTTATTGTAAGCGACAAA**TCTAGA**

Main promoters:

appYp (position -114 from the ATG)

Positive selection

```
Initial TAAAAAACAACATACATATAATAATTTAATCTTAAATGAAATTTATTAAAATTTGCAAaCTATAATTTT
cycle_1 TAAAAAACAACATACATATAATAATTTAATCTTAAATGAAGTTTATTAAAATTTGCAAaCTATAATTTT
cycle_2 TAAAAAACAACATACATATAATAATTTAATCTTAAATGAAGTCTATTAAAATTTGCAAaCTATAATTTT
cycle_3 TAAAAAACAACATACATATAATAATTTAATCTTAAATGAAGTCTATTAAAATTTGCAAaCTATAATTTT
cycle_4 TAAGAAACAACATACATATAACAATTTAATCTTAAATGAAGTCTATTAAAATTTGCAAaCTATAAATTT
cycle_5 TAAGACTCAACATACATATAACAATTTAATCTTAAATGAAGTCTATTAAAATTTGCAAaCTAACAATTT
cycle_6 TAAGACTCAACATACATATAACAATTTAATCTTAAATGAAGTCTATTAGAATTTGCAAaCAAACAATTT
cycle_7 TAAGACGCGACATACATATAACAATTTAATCTTAAATGTAGTCTATTAGAATTTGCAAaCAAACAATTT
cycle_8 TAAGACGCGACATACATATAACAATTTAATCTTAAATGTAGTCTATTAGCATTTGCAAaCAAACAATTT
cycle_9 TAAGACGCGACATACATATAACAATTTAATCTTAAATGTAGTCTATTAGCATTTGCAAaCAAACAATTT
                                  -35                   -10      TSP
```

Negative selection

```
Initial TAAAAAACAACATACATATAATAATTTAATCTTAAATGAAATTTATTAAAATTTGCAAaCTATAATTTT
cycle_1 TAAAAAACAACATACATATAATAATTTAATCTTAAATGAAATTTATTAAAATCTGCAAaCTATAATTTT
cycle_2 TAAAAAACAACATACATATATTAATTTAATCCTAAATGAAATTTATTAAAATCTGCAAaCTATAATTTT
cycle_3 TAAAAAACAACATACATATATTAATTTAATCCTAAATGAAATTTATTAAAATCTGCAAaCTATAATTTT
                                  -35                   -10      TSP
```

Pσ38 (position -25 from the ATG)

Positive selection

```
Initial ACATCATCCTGATTATGATTGTGTATTTAATTGGTTGTTATTTGACTACTATCAACTTgTTTTAATTTT
cycle_1 ACATTATCCTGATTGTGATTGTGTATTTAATTGGTTGTTATTTGACTACTATCAACTTgTTTTAATTTT
cycle_2 ACATTATCCTGATTGTGATTGTGTATTTAATTGGTTGTTATTTGACTACTATCAACTTgTTTTAATTTT
cycle_3 ACATTATCCTGATTGTGATTGTGTATTTAATTGGTTGTTATTTGACTACTACCAACTTgTTTTAATTTT
cycle_4 ACATTATCCTGATTGTGATTGTGTATTTAATTGGTTGTTATTTGACTACTACCAACTTgGTTTAATTTT
cycle_5 ACATTATCCTGATTGTGATTGTGTATTTAATTGGTTGTTATTTGACTACTACCAACTAgGTTTAATCTT
cycle_6 ACATTATCCTGATTGTGATTGTGTATTTAACTGGTTCTTATTTGACTACTACCAACTAgGTTTAGTCTT
cycle_7 ACATCATCCTGATTGTGATTGTGTATTTAACTGGTTCTTATATGACTACTGCCAACTAgGTTTAGTCTT
cycle_8 ACATCATCCTGATTGTGATTGTGTATTTAACTGGTTCTTATATGACTACTGCCAACTAgGTTTAGTCTT
cycle_9 ACATCATCCTGATTGTGATTGTGTATTTAACTGGTTCTTATATGACTACTACCAACTAgGTTTAGTCTT
                                     -35                -10      TSP
```

Negative selection

```
Initial ACATCATCCTGATTATGATTGTGTATTTAATTGGTTGTTATTTGACTACTATCAACTTgTTTTAATTTT
cycle_1 ACATCATCCTGATTATGATTGTGTATTTAATTGGTTGTTATTTGACTACTATCAACTTgTTTTAATTTT
cycle_2 ACATCATCCTGATTATGATTGTGTATTTAATTGGTTGTTATTTGACTACTATCAACTTgTTTTAATTTT
cycle_3 ACATCATCCTGATTATGATTGTGTATTTAATTGGTTGTTATTTGACTACTATCAACTTgTTTTAATTTT
                                    -35                 -10      TSP
```

P-85 (position -85 from the ATG)

Positive selection

```
Initial TCTTAAATGAAATTTATTAAAATTTGCAAACTATAATTTTGTGTATAAAAATATAAATgCACATCATCC
cycle_1 TCTTAAATGAAGTTTATTAAAATTTGCAAACTATAATTTTGTGTATAAAAATATAAATgCACATTATCC
cycle_2 TCTTAAATGAAGTCTATTAAAATTTGCAAACTATAATTTTGTGTATAAAAATATAAATgCACATTATCC
cycle_3 TCTTAAATGAAGTCTATTAAAATTTGCAAACTATAAATTTGTGTAT-AAACTATAAATgCACATTATCC
cycle_4 TCTTAAATGAAGTCTATTAAAATTTGCAAACTATAAATTTGTGTAT-AAACTATAAATgCACATTATCC
cycle_5 TCTTAAATGAAGTCTATTAAAATTTGCAAACTAACAATTTGCGTAT-ATACTATAAATgCACATTATCC
cycle_6 TCTTAAATGAAGTCTATTAGAATTTGCAAACAAACAATTTGCGTAT-ATACTATAAATgCACATTATCC
cycle_7 TCTTAAATGTAGTCTATTAGAATTTGCAAACAAACAATTTGCGTAT-ATACTATAAATgCACATCATCC
cycle_8 TCTTAAATGTAGTCTATTAGCATTTGCAAACAAACAATTTGCGTAT-ATACTATAAATgCACATCATCC
cycle_9 TCTTAAATGTAGTCTATTAGCATTTGCAAACAAACAATTTGCGTAT-ATACTATAAATgCACATCATCC
                                  -35                 -10        TSP
```

Negative selection

```
Initial TCTTAAATGAAATTTATTAAAATTTGCAAACTATAATTTTGTGTATAAAAATATAAATgCACATCATCC
cycle_1 TCTTAAATGAAATTTATTAAAATCTGCAAACTATAATTTTGTGTATAAAAATATAAATgCACATCATCC
cycle_2 TCCTAAATGAAATTTATTAAAATCTGCAAACTATAATTTTGTGTATAAAAATATAAATgCACATCATCC
cycle_3 TCCTAAATGAAATTTATTAAAATCTGCAAACTATAATTTTGTGTATAAAAATATAAATgCACATCATCC
                                 -35                  -10        TSP
```
